# Case Report: Recurrent pulmonary infections with neck masses: a diagnostic challenge and literature review

**DOI:** 10.3389/fmed.2026.1807946

**Published:** 2026-04-10

**Authors:** Leilei Zhao, Jianshe Zhao, Yi Lu

**Affiliations:** Jinan Children’s Hospital, Jinan, China

**Keywords:** APDs, child, immunity, infectious mononucleosis, lungs

## Abstract

This article presents a case characterized by recurrent pulmonary infections and a poor therapeutic response. Initially, cystic fibrosis (CF) or Primary ciliary dyskinesia was suspected; however, the patient subsequently sought medical attention for a neck mass. The mass was initially misdiagnosed as lymphoma, but a comprehensive examination and assessment ultimately confirmed the diagnosis of activated phosphoinositide 3-kinase delta syndrome type I(APDS1). Through the patient’s tortuous diagnostic journey, this report aims to enhance clinicians’ understanding of this rare primary immunodeficiency disorder. Clinical picture: The patient is a female adopted child who has experienced recurrent lung infections since the age of 6 months. On average, she has required hospitalization 2–3 times annually, with conventional anti-infection treatments proving less effective. Throughout her illness, she was admitted to another hospital for sinusitis and breast cysts, although the specifics of her treatment remain unclear. Subsequently, she presented to our hospital with symptoms of coughing, yellow sputum, and hemoptysis. During her hospitalization, a new neck mass was identified. Key inspection: Imaging assessment revealed a chest CT indicating pulmonary infection and bronchiectasis with mucus plug formation. Neck CT and MRI suggested a potential diagnosis of lymphoma. Pathogen testing through nucleic acid analysis for respiratory pathogens yielded positive results for mycoplasma and adenovirus influenzae. Laboratory and abdominal ultrasound did not identify any abnormalities in the pancreas. A peripheral blood immunological assessment demonstrated significantly diminished cellular and humoral immune functions. Following the resection of the cervical mass, the initial pathological diagnosis was lymphoma; however, tests for T-cell receptor (TCR) and immunoglobulin (IG) gene rearrangements returned negative results. After a multidisciplinary consultation and subsequent pathological re-examination, the diagnosis was revised to infectious mononucleosis. Genetic testing revealed a heterozygous mutation in the PIK3CD gene. Final diagnosis: Activated phosphatidylinositol 3-kinase δ syndrome type I (APDS1). Treatment and prognosis: Following diagnosis, the child was administered rapamycin-targeted therapy in conjunction with compound sulfamethoxazole to prevent infections, with regular monitoring of rapamycin blood concentrations. Subsequent follow-up indicated a significant reduction in the frequency of pulmonary infection episodes, a decrease in the size of the enlarged lymph nodes in the neck, and effective control of the condition.

## 1 Introduction

Activated phosphoinositide 3-kinase delta syndrome (APDS) is a rare PIDD caused by gain-of-function (GOF) mutations in PIK3CD (APDS1) or PIK3R1 (APDS2) genes, which was first reported in 2013 (1). The core pathogenesis is the hyperactivation of PI3Kδ-AKT-mTOR signaling pathway, leading to combined cellular and humoral immune dysfunction.(2). The phenotypes of APDS are highly heterogeneous (3, 4): Ranging from asymptomatic or subclinical infection, to serious immunodeficiency. The majority of patients present with recurrent sinopulmonary infections, elevated levels of serum immunoglobulin (IgM), low levels of serum IgG, lymphoproliferation, autoin flammatory disease, Epstein-Barr virus (EBV) and cytomegalovirus (CMV) viremia, as well as Epstein-Barr virus (EBV) and non-EBV driven malignancies. Almost all patients have benign lymphoid tissue hyperplasia (e.g., hepatosplenomegaly and lymph node enlargement). No recent epidemiological review on APDS was identified. However, a recently published study by Vanselow et al. estimated that approximately 1∼2 individuals per million population are affected by APDS (5). Diagnosis of APDS remains challenging due to the relatively short time since the disease was recognized, limited disease awareness, and its highly heterogeneous clinical manifestations, leading to a low diagnostic rate. As understanding of the disease improves and relevant evidence accumulates, the estimated prevalence of APDS is expected to rise. APDS1 is the most common subtype, accounting for about 73.6% of all APDS cases, and is caused by heterozygous mutations in PIK3CD gene encoding the p110δ catalytic subunit (6). The classic clinical manifestations of pediatric APDS1 include recurrent pneumonia, sinusitis, bronchiectasis, generalized lymphadenopathy and splenomegaly. Respiratory tract infections (65%) were the most common first presentation in APDS patients followed by organomegaly. Bronchiectasis emerged as a common complication resulting from lung infections, impacting approximately 60% of patients. Approximately, about 75% patients with APDS presenting as lymphadenopathy and nodular lymphoid hyperplasia,13% of APDS patients were diagnosed with lymphoma including both Hodgkin and non-Hodgkin’s lymphoma (6). Intriguingly, a considerable proportion of these lymphoma cases exhibit an association with EBV infection (6, 7). These symptoms overlap significantly with cystic fibrosis, primary ciliary dyskinesia, and lymphoproliferative disorders, leading to frequent misdiagnosis in the early stage. In recent years, with the development of genetic testing technology and the update of clinical guidelines, the diagnostic awareness of APDS has been continuously improved, and the targeted treatment options have been enriched. However, for special populations such as adopted children without family genetic information, the diagnosis is still challenging due to the lack of family history clues. This study reports an adopted pediatric APDS1 case with recurrent pulmonary infections as the initial manifestation and neck mass as the secondary main complaint, which was successively suspected of CF/PCD and misdiagnosed as lymphoma. By combining the latest 2024–2025 APDS diagnostic criteria and treatment progress (4, 8, 9), the diagnostic pitfalls and clinical management of this case were analyzed, aiming to improve the clinical recognition of pediatric APDS1 and reduce the rate of misdiagnosis and delayed treatment.

## 2 Case presentation

### 2.1 General information

A 13-year-old female adopted child was admitted to our hospital in March 2024 due to “hemoptysis for 3 days.” The patient was adopted from a welfare home, and the parental genetic information and family medical history were unknown. The past medical history was provided by the adoptive parents, and the patient had no history of allergies or autoimmune diseases.

### 2.2 Clinical course

#### 2.2.1 Chronic recurrent respiratory infections (6 months to February 2024)

The patient had recurrent pneumonia and bronchitis since 6 months of age, with 2–3 hospitalizations per year, and the curative effect of conventional anti-infection treatment was poor. In 2019, she was diagnosed with sinusitis in Our hospital’s outpatient department. In 2023, she underwent breast cyst resection (pathological results were unknown). In January-February 2024, she was treated in an external hospital for severe pneumonia complicated with lung consolidation and bronchiectasis, and the pathogenic examination showed co-infection with fungi, Haemophilus influenzae, adenovirus and streptococcus.

#### 2.2.2 First admission (March 2024): hemoptysis with suspected CF/PCD

The patient developed severe cough during eating 3 days before admission, followed by fresh hemoptysis (4 mouthfuls within 30 min, about 5 mL each time) without other obvious discomfort.

Admission diagnoses: Hemoptysis (undiagnosed etiology); Suspected PCD; Sinusitis.

Key examinations: ➀ Routine blood test: Mild hypochromic anemia (Hb 113.0 g/L), decreased lymphocyte count (0.98 × 10^9^/L) and ratio (12.5%). ➁ Cytokines: IL-10 (2.16 pg/mL) and IFN-γ (0.46 pg/mL) were decreased. ➂ Lymphocyte subsets: Significant reduction in the count of total T cells, helper/inducer T lymphocytes, natural killer (NK) cells, suppressor/cytotoxic T lymphocytes, B lymphocytes (all lower than the lower limit of the pediatric reference range). ➃ Imaging: Chest CT showed pulmonary infection, bronchiectasis with mucus plug formation and bronchial artery dilatation. Sinus CT confirmed sinusitis; Abdominal ultrasound showed splenomegaly and normal pancreatic function (excluding CF). ➄ Pathogen detection: Respiratory pathogen nucleic acid test was positive for mycoplasma and adenovirus.

Treatment and discharge: Bronchial artery embolization was performed on March 17, and hemoptysis was completely relieved. Anti-infection, antitussive, expectorant and immunoregulatory treatments were given. Genetic testing samples were collected on March 23.

Discharge diagnoses: Hemoptysis; Bronchiectasis with infection. Pneumonia with consolidation; Suspected PCD. Right neck mass (undiagnosed etiology); Splenomegaly.

#### 2.2.3 Second admission (April 2024): neck mass with suspected lymphoma

The patient was re-admitted due to progressive enlargement of the right neck mass (no tenderness).

Admission diagnosis: Neck mass (suspected lymphoma).

Key examinations: ➀ Immunoglobulin: IgM was significantly elevated (8.32 g/L, reference range 0.42–1.46 g/L), IgA and IgG were normal. ➁ EBV detection: EBV-IgM was suspicious (30.9 U/mL on April 7, 23.8 U/mL on April 8), EBV-DNA was negative (< 10^3^ copy/mL). ➂ Imaging: Cervical CT/MRI showed globular enlargement of bilateral cervical lymph nodes (predominantly on the right) with heterogeneous enhancement and no surrounding inflammatory exudation; PET-CT showed increased FDG metabolism in nasopharyngeal wall, tonsils, thymus and spleen with corresponding enlargement. ➃ Pathological examination: Initial pathological diagnosis of right neck mass resection was nodular lymphocyte-predominant Hodgkin lymphoma (NLPHL); Re-examination by Beijing GoBroad Boren Hospital combined with TCR/IG gene rearrangement (negative) confirmed IM associated with EBV infection (EBER+, EBNA2+, LMP1+). ➄ Bone marrow biopsy and cerebrospinal fluid examination: No pathological cells were found. ➅ Genetic testing: A heterozygous missense variant of “PIK3CD” gene (c.3061G > A, p.Glu1021Lys, NM_005026.5) was detected, which was classified as Pathogenic according to ACMG guidelines (PS4_VeryStrong, PP1_Strong, PS3, PM2_Supporting) and recorded in ClinVar (ID: 88675) as a classic pathogenic mutation of APDS1.

Multidisciplinary consultation (April 26): Combined with the clinical manifestations (recurrent pulmonary infections, lymphoproliferation), immunological abnormalities (combined cellular and humoral immune dysfunction, elevated IgM), genetic testing results and pathological re-examination results, the final diagnosis was confirmed as APDS1 complicated with EBV-associated IM.

#### 2.2.4 Treatment and follow-up

Targeted treatment regimen: Oral sirolimus (0.64 mL q12h, based on body weight 40 kg) was initiated on April 27, combined with trimethoprim-sulfamethoxazole (TMP-SMX) for infection prophylaxis, regular immunoglobulin replacement therapy and anti-infection treatment for 2–3 weeks. Therapeutic drug monitoring (TDM) of sirolimus blood concentration was performed regularly, and the dosage was adjusted according to the results.

**FIGURE 1:**
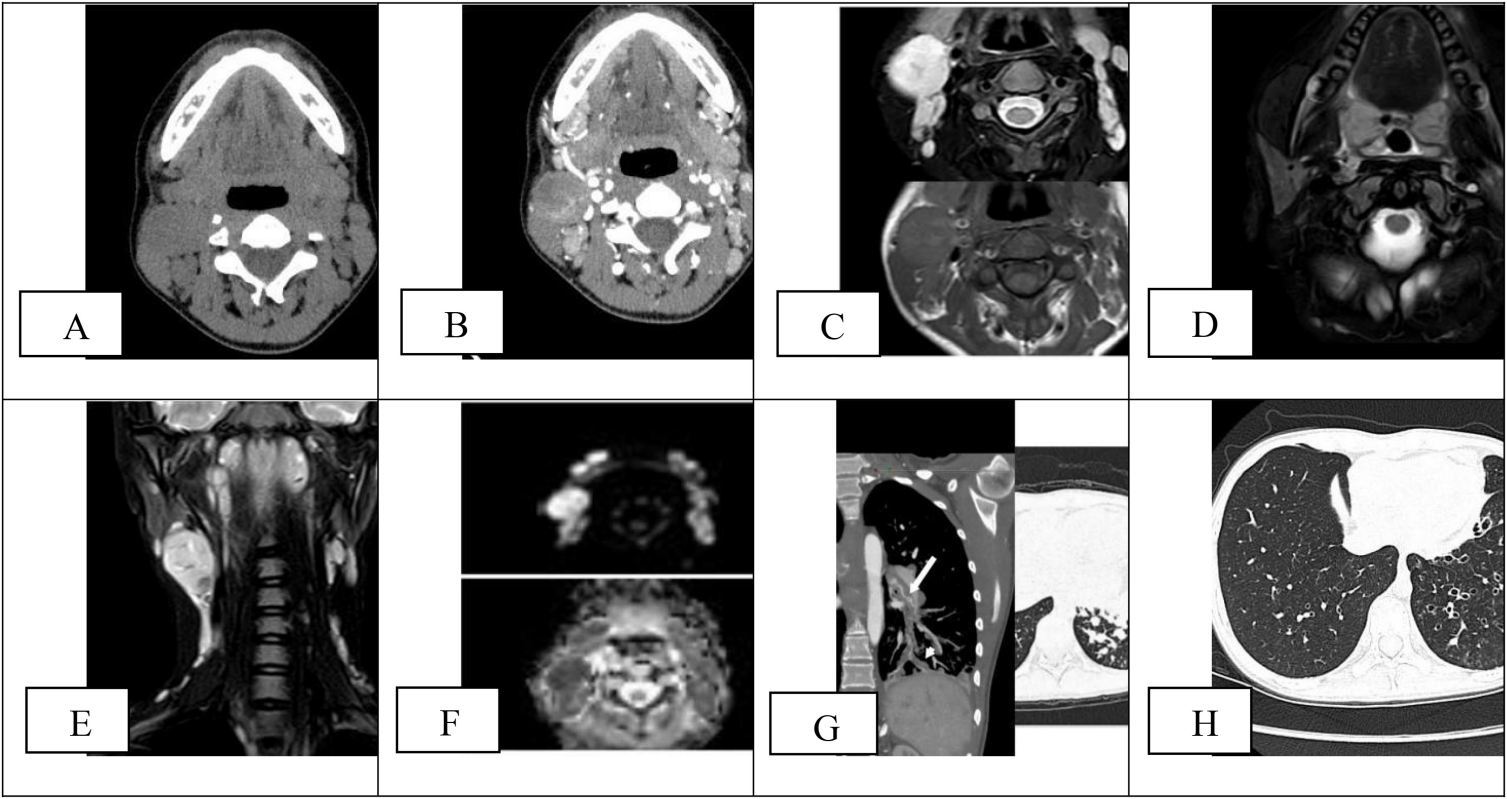
**(A,B)** Multiple enlarged lymph nodes in bilateral cervical regions, predominantly on the right side. The lymph node shows abnormal morphology, are globularly enlarged, and demonstrate heterogeneous enhancement. The surrounding fat planes are well-defined, with no obvious exudation. **(C–F)** MRI T2-weighted images demonstrated focal nodular mucosal thickening in the right pharyngeal recess, with restricted diffusion on DWI. Bilateral cervical lymphadenopathy, predominantly right-sided, showed heterogeneous T2 signal without infiltration of surrounding fat. **(G,H)** Showed: multiple bronchodilatation, with mucusclots (short arrow) and bronchial artery dilation (long arrow). (**G**, March 16, 2024); Re-examination after treatment with Sirolimus. The effect is remarkable (**H**, January 25, 2026).

Follow-up outcomes (April 2024 to January 2026): ➀ The frequency of pulmonary infections was significantly reduced, and no severe pneumonia occurred during follow-up. ➁ Chest CT re-examination (January 25, 2026) showed significant improvement of bronchiectasis and disappearance of mucus plug (Figure 1H). ➂ Cervical lymphadenopathy was significantly reduced, and no progressive enlargement was found. ➃ Hematological indicators: The three lineages of blood cells returned to normal in August 2024. ➄ Adverse reactions: Mild vomiting occurred in November 2024 (self-discontinued medication for 1 week). Irregular medication due to personal reasons.

### 2.3 Auxiliary examination results

**FIGURE 2:**
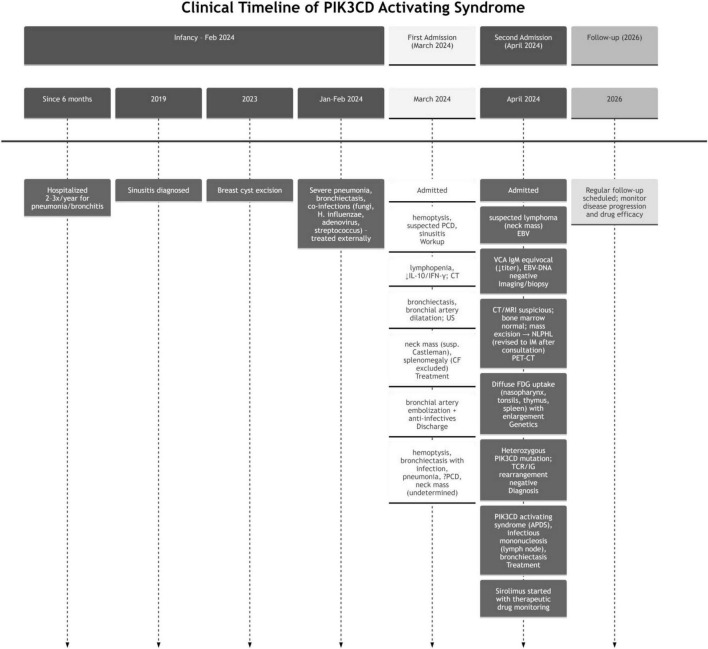
Clinical timeline of PIK3CD activating syndrome.

**FIGURE 3:**
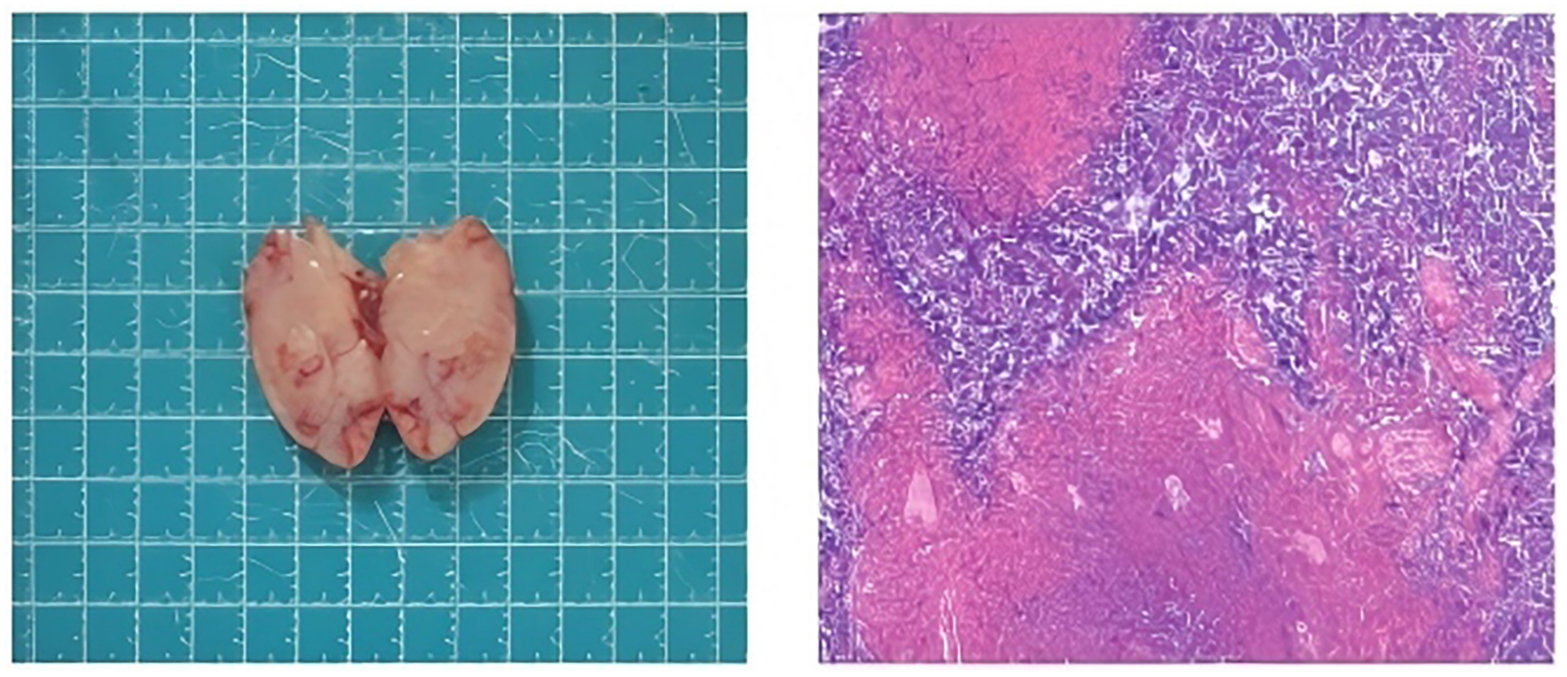
Gross description: One nodule measuring. The surface is smooth and grayish-red with an intact capsule. The cut surface is grayish-white to grayish-red, soft and fine in texture, with focal grayish-yellow areas suggestive of necrosis. Microscopic Features: A monomorphous population of intermediate-sized cells is noted, with moderate amount of cytoplasm, oval to slightly irregular nuclei, fine granular chromatin, and indistinct nucleoli. Scattered larger cells with prominent nucleoli are present. The background contains plasma cells and abundant large histiocytes with abundant cytoplasm, showing phagocytic activity. Immunohistochemistry: CD3(-), CD20(+), CD15(-), CD21(FDC+), CD30(+), ALK(-), EMA(-), CD19(+), Oct-2(+), Bob1(+), CD45(+), MUM-1(+), TdT(-), PAX-5(+), c-myc(-), Ki-67(+, 85%), Bcl-2(+), Bcl-6(+). In situhybridization: 2024-08638-4:EBER(+ ). Immunohistochemistry reassessment: BCL-2(+), BCL-6(+), CD3(+), numerous, CD20 (focal+), CD15(-), CD-21(FDC+), CD30(scattered), ALK(-), CD45(+ ), MUM1(plasma cells + ), c-myc (< 5% + ), Ki-67(+); proliferative ind-ex70%), EMA(-), CD19(focal+). In situhybridization EBER(+), scattered positive cells in both small and large cells. Special studiesCD4(+ ), fewer than CD8,CD8(+), numerous, EBNA2(+), LMP1(+), CD163(+), numerous.

See Table 1 (Laboratory studies), Table 2 (Genetic testing), Table 3 (Monitoring of rapamycin blood drug concentration), Figure 2 (Clinical timeline), Figure 1 (Imaging findings), Figure 3 (Pathological features) for detailed results.

**TABLE 1:** Laboratory studies.

Inspection time	Item name	Result	Reference range
March 16, 2024	Blood routine examination	WBC 7.83 × 10^9^/L	4.3∼11.3 × 10^9^/L
		RBC 4.73 × 10^12^/L	4.2∼5.7 × 10^12^/L
		HB 113.0g/L	118∼156g/L
		CRP 7.29 mg/L	<20 mg/L
		ESR 21 mm/h	0∼20 mm/h
		Lymphocyte count 0.9800 × 10^9^/L	1.5∼4.610^9^/L
		lymphocyte ratio12.5%	23%∼59%
March 16, 2024	Six types of cytokines	Leukocyte factor-10 2.1,600 pg/mL	2.60∼4.90 pg/mL
		Interferon∼γ 0.4600 pg/mL	1.60∼17.30 pg/mL
March 17, 2024	Absolute count of lymphocyte subsets	Count of total T cells 620.000	1297∼2,480 one/μL
		Relative count of helper/inducer T cells 41.8500	28.47∼41.39%
		Count of helper/inducer T lymphocytes349.000	621∼1258 one/μL
		Count of suppressor/cytotoxic T lymphocytes 217.0000	509∼1050 one/μL
		Count of natural killer (NK) cells 38.0000	203∼584 one/μL
		Count of B lymphocytes 147.0000	247∼578 one/μL
April 07, 2024	Immunoglobulin	IgA:1.0800 g/L	0.63∼3.04 g/L
		IgM:8.3200 g/L	0.42∼1.46 g/L
		IgG:9.1600 g/L	7.1∼15.6 g/L
April 07, 2024	EBV∼Ab	EBV∼IgM 30.9U/mL	Negative<20 U/mL
			Suspicious 20∼40 U/mL
			Positive>40
April 08, 2024	EBV∼Ab	EBV∼IgM 23.8U/mL	Negative<20 U/mL
			Suspicious 20∼40 U/mL
			Positive>40
April 08, 2024	EBV∼DNA	<10^3^copy/mL	<10^3^ copy/mL

**TABLE 2:** Genetic testing.

Gene	Chromosome location	Reference transcript	HGVS notation (DNA/protein)	Zygosity	ACMG classification	Functional effect	ClinVar ID	Parental testing
PIK3CD	chr1:9787030	NM_005026.5	c.3061G > A (p.Glu1021Lys)	Heterozygous	Pathogenic	Gain-of-function	88675	Not performed

**TABLE 3:** Monitoring of rapamycin blood drug concentration.

Date	Test result (ng/mL)	Medication and clinical notes
2024-04-27	–	Started oral administration: 0.64 mL, q12 h
2024-05-02	9.1	Follow-up
2024-05-23	3.7	Follow-up
2024-06-20	2.5	Follow-up
2024-07-16	4.2	Follow-up
2024-08-08	0.6	Follow-up; blood counts (three lineages) normalized
2024-11-14	–	Follow-up; obvious vomiting, self-discontinued meds for 1 week
2025-03-01	–	Follow-up; irregular medication due to personal reasons

## 3 Discussion

This case is a typical pediatric APDS1 with adopted child without family history, recurrent pulmonary infections as the initial manifestation and EBV-associated IM as the secondary manifestation, which experienced the diagnostic process of “suspected CF/PCD → suspected lymphoma → confirmed APDS1”. In light of recent advances in APDS research, the key diagnostic, differential diagnostic, and therapeutic issues of this case are discussed below.

### 3.1 Diagnostic pitfalls of pediatric APDS1: overlapping clinical manifestations with CF/PCD

Recurrent respiratory infections and bronchiectasis are the core initial manifestations of pediatric APDS1, with a prevalence of 65 and 60% respectively (6), which are highly overlapping with CF and PCD, leading to easy misdiagnosis in the early stage. In the study of Adam et al., it report 8 people from 5 countries with chronic upper and lower respiratory symptoms highly suggestive of PCD who were found to have APDS1 as the result of heterozygous, pathogenic, acti vating PIK3CD variants (10). The key differential points are as follows: ➀ Pathogenesis: APDS1 is an immunodeficiency disease, while CF is a disorder of ion transport function and PCD is a disorder of ciliary motile function (11, 12). ➀ Systemic manifestations: CF is often accompanied by pancreatic insufficiency, steatorrhea and meconium ileus (12), and PCD is often associated with Kartagener syndrome (situs inversus, sinusitis, bronchiectasis) (13), while APDS1 is often accompanied by lymphoproliferation, elevated IgM and splenomegaly (4). ➀ Imaging features: APDS1-related bronchiectasis is mostly secondary to recurrent infections, with uneven distribution and often accompanied by lymphadenopathy (14, 15). The patients with PCD, bronchiectasis predominantly involves the middle lobes, whereas in cystic fibrosis, it mainly affects the upper lobes or is diffusely distributed throughout both lungs (16). In this case, the pancreatic function was normal (excluding CF), and there was no situs inversus (excluding classic Kartagener syndrome), but PCD was still initially suspected due to the lack of awareness of APDS1. Therefore, for children with recurrent respiratory infections and bronchiectasis, if conventional anti-infective treatment is ineffective and is accompanied by immune function abnormalities (including lymphopenia and abnormal immunoglobulin levels), genetic testing for APDS-related genes should be performed in a timely manner to avoid misdiagnosis.

### 3.2 lymphoproliferation in APDS1: misdiagnosis risk of lymphoma and the core role of multidisciplinary

Non-neoplastic lymphoproliferative disorders or malignant tumors are typical manifestations of activated PI3Kδ syndrome (APDS). APDS patients have an increased risk of malignant transformation (predominantly hematological malignancies) due to their inherent lymphoproliferative tendency. Pathogenetically, PI3Kδ hyperactivation and defective EBV immune surveillance are key to lymphomagenesis in this population. The prevalence of malignancy is as high as 30% in APDS1,with Hodgkin lymphoma and diffuse large B-cell lymphoma being the most prevalent (17). Multiple studies indicate that impaired Epstein-Barr virus (EBV) clearance and intrinsic B-cell proliferation contribute to lymphomagenesis. A study by Mattia Moratti et al. on 85 LPD patients showed that 45% (38/85) were ultimately diagnosed with Inborn errors of immunity (IEI). Among genetically confirmed IEI cases, 24% progressed to lymphoma, with APDS patients having the highest malignant transformation risk (40%) and a later onset age. Notably, lymphoproliferative lesions in immunodeficient patients often mimic malignant disorders. A 31% of patients initially diagnosed with hematologic malignancies were reclassified as nonneoplastic/reactive after IEI diagnosis and histologic reassessment (18). Radiological features made distinguishing lymphoma from reactive lesions challenging. These findings highlight the necessity of early immunologic and genetic evaluation in pediatric LPD patients to avoid misdiagnosis and guide precision management.

In the present case, the pathological diagnosis of the lymph node lesions was controversial between lymphoma and infectious mononucleosis (IM): Immunohistochemical analysis revealed strong B-cell marker expression (CD20, PAX5, Oct2, BOB.1), CD45^+^, CD15^–^, CD21^+^ (preserved nodular architecture), and BCL-6^+^, initially supporting NLPHL. However, concurrent high Ki-67 expression and CD30 positivity (atypical for NLPHL) and subsequent findings (markedly elevated Ki-67, EBER^+^, polyclonal lymphoproliferation confirmed by negative TCR/IG gene rearrangement) confirmed the final diagnosis of IM (19–27). Accurate identification of APDS-related lymphoproliferative lesions is crucial for subsequent treatment.

The management of APDS includes antibiotic prophylaxis, immunoglobulin replacement therapy, targeted immunosuppressive agents, and hematopoietic stem cell transplantation (HSCT). Sirolimus (rapamycin) and leniolisib (CDZ173) are commonly used targeted therapeutic agents. Sirolimus alleviates benign lymphoproliferation, improves immune parameters and natural killer (NK) cell function, but exhibits limited efficacy in treating cytopenia and gastrointestinal inflammation; long-term adverse effects require close monitoring (5, 28–32). Leniolisib normalizes transitional and naïve B cells, reduces senescent T cells and serum IgM levels, and decreases lymph node and spleen size with favorable tolerability (33, 34). In a study by Federica Barzaghi et al., rapamycin, administered in 36% of patients, demonstrated efficacy in controlling lymphoproliferation. In contrast, the selective PI3Kδ inhibitor leniolisib, used in 32% of patients, conferred benefits in both infection control and the amelioration of immune dysregulation. Additionally, three patients underwent successful HSCT due to recurrent infections despite ongoing prophylaxis or lymphoproliferation that was poorly responsive to rapamycin (35).

However, in China, only sirolimus has been approved for clinical use, while leniolisib has not yet been approved. After the diagnosis of this child was confirmed, unnecessary chemotherapy was avoided. Following the administration of immunoglobulin replacement therapy, anti-infection treatment, and rapamycin therapy, although the pulmonary infection was effectively controlled and lymphadenopathy improved, the patient still took medication irregularly and intermittently due to adverse reactions such as vomiting. Therefore, long-term follow-up is necessary to promptly adjust the patient’s medication regimen and monitor therapeutic efficacy.

## 4 Patient perspective

We traveled to many hospitals to seek treatment for our child, experiencing hardships and confusion, but we still failed to obtain a clear diagnosis and effective treatment. It was not until we came to your hospital that, under the careful examination and standardized diagnosis and treatment of the medical team, our child finally got a clear diagnosis and targeted effective treatment. We are very satisfied with the rigorous examination process, efficient diagnosis and treatment efficiency of the doctors in the hospital, as well as the obvious improvement of our child’s condition after treatment, and we sincerely thank all the medical staff for their professionalism and dedication.

## 5 Conclusion

Activated PI3K delta syndrome (APDS) is a rare primary immunodeficiency resulting from gain-of-function (GOF) mutations in PIK3CD or PIK3R1, leading to immune dysregulation. It typically manifests as recurrent respiratory infections, lymphoproliferation, autoimmunity, and an increased risk of malignancy. Given the significant overlap in clinical manifestations with other diseases, insufficient familiarity with APDS may easily result in misdiagnosis as multiple other independent disorders and delayed treatment. Before reaching 11 years of age, the patient in this case primarily displayed pulmonary symptoms such as recurrent pneumonia, sinusitis, and bronchiectasis. The patient received care from multiple institutions with inconsistent follow-up, and the clinical features overlapped with those of other pulmonary conditions, posing challenges in establishing an early diagnosis. A multidisciplinary approach that integrates a detailed medical history, advanced imaging, and molecular diagnostics (including PI3Kδ pathway analysis) is crucial for prompt and precise diagnosis, facilitating targeted treatment, and ultimately enhancing patient outcomes. Meanwhile, for adopted children with a long history of recurrent lung infections, early genetic testing is crucial to rule out primary immunodeficiency disorders.

## Data Availability

The original contributions presented in the study are included in the article/supplementary material, further inquiries can be directed to the corresponding authors.
